# Mutational features of chromids and chromosomes in *Pseudoalteromonas* provide new insights into the evolution of secondary replicons

**DOI:** 10.1128/spectrum.02127-24

**Published:** 2025-03-25

**Authors:** Wanyue Jiang, Jiao Pan, Tongtong Lin, Yaohai Wang, Yanze Wang, Rongxiao Zhang, Xiaoming Zhou, Yu Zhang

**Affiliations:** 1 Key Laboratory of Evolution and Marine Biodiversity (Ministry of Education), Institute of Evolution and Marine Biodiversity, KLMME, Ocean University of China 535359 , Qingdao, Shandong, China; 2 Laboratory for Marine Biology and Biotechnology, Qingdao Marine Science and Technology Center, Qingdao, Shandong, China; 3 School of Mathematics Science, Ocean University of China 12591, Qingdao, Shandong, China; University of Pittsburgh, Pittsburgh, Pennsylvania, USA

**Keywords:** multi-replicon, mutation features, replication direction, chromid evolution, *Pseudoalteromonas*

## Abstract

The genomes of multi-replicon bacteria are composed of a primary replicon (the chromosome) and secondary replicons (chromids). Currently, there is a lack of understanding of the mutation features and evolutionary patterns of these different replicons. Specifically, in the genus *Pseudoalteromonas*, the chromids of multi-replicon species exhibit both unidirectional and bidirectional replication. Here, we investigated the similarities and differences between chromosomes and chromids in sequence composition and gene synteny of *Pseudoalteromonas* species by comparative genomic analysis, as well as the spontaneous mutation features of different replicons by mutation accumulation (MA) experiments combined with whole-genome sequencing strategy (MA-WGS). MA-WGS analysis revealed that there was no significant difference between chromids and chromosomes in the mutation rate or mutation spectrum of *P*. sp. LC0214 (where the chromid is unidirectional in replication) and *P*. sp. JCM12884^T^ (where the chromid is bidirectional in replication). In addition, the context-dependence and variation pattern of the base-pair substitutions (BPSs) rates of the entire replicons exhibited differences that may be caused by the different replication directions of the chromids. The results of this study provide a new theoretical foundation for an in-depth understanding of the origin and evolution of chromids in multi-replicon bacterial species and facilitate further exploration of the complex mechanisms of bacterial diversity.

**IMPORTANCE:**

*De novo* mutations are a critical driving force in species evolution. Currently, there is a lack of sufficient research on the influence of replicon types on the occurrence of genomic mutations in bacteria. Moreover, the scarcity in systematic analysis and comparison of spontaneous mutation features between different replicons results in the limited information on the evolutionary dynamics of multi-replicon species. The diversity of replication direction in the multi-replicon species of the genus *Pseudoalteromonas* provides a unique opportunity for studying the impact of replication direction on the patterns of mutation. In addition to the composition characteristics between chromosomes and chromids, the spontaneous mutation rates in the context-dependence and variation pattern of the base-pair substitutions (BPSs) across different replicons within *Pseudoalteromonas* species revealed in this study provide valuable insights into the evolutionary dynamics of bacterial secondary replicons.

## INTRODUCTION

Among bacteria with published genomic information, about 10% are multi-replicon species, whose genomes mainly contain primary replicon (i.e., the chromosome) and secondary replicons (1, 2). Generally, these secondary replicons carry genes essential for the growth of the organism, forming an integral part of the genome. Secondary replicons are diverse, where chromids, as distinct from chromosomes and plasmids, are significantly more prevalent (2). One of the essential differences between chromids and chromosomes is that chromids have a plasmid-type replication system (1–8). The hypothesis that chromids originated from the evolutionary precursor megaplasmids almost certainly represents the mechanism of formation of basic secondary replicons in most species studied to date (1, 9–15).

It has been reported that the evolutionary pattern and genetic mutation rate of replicons are unique in multi-replicon species. For example, in *Sinorhizobium meliloti*, the chromosome is vertically transmitted and structurally stable, while the chromid was formed by ancient horizontal gene transfer and was subject to greater positive selection (16). Similarly, in some other multi-replicon genera, genes located on chromosomes are more conserved between species than those located on chromids, and genes located on megaplasmids or smaller chromids are less conserved (17–20). In addition, it has been reported that chromid orthologous sequences evolve faster than the corresponding chromosome orthologous sequences (21). These increased evolutionary rates of chromids appear to be due to weakened selection for the use of specific codons and translation efficiency, resulting from decreased gene expression frequency or necessity (11, 21–25). In multi-replicon bacterial species, replication bias may also accelerate the evolution of secondary replicons (26). The above investigations indicate that each replicon in the genome of multi-replicon species may experience different evolutionary rates and selection pressures, and that chromosomes are the most genetically stable replicons, followed by chromids. However, most of the above studies on the evolutionary characteristics of replicons have only focused on partial sequences or gene levels in the genome. At the whole-genome level, there is still a lack of research on the spontaneous mutation features and evolutionary patterns of different types of replicons, which has hindered an in-depth understanding of the origin and the evolution of bacterial replicons.

Multi-replicon genomes, which include plant symbionts such as the nitrogen-fixing *Rhizobia* and several kinds of pathogens, including the genera *Brucella*, *Vibrio*, and *Burkholderia* as well as the marine microbial *Pseudoalteromonas*, are scattered throughout the bacterial species phylogenetic tree (2). Single-replicon species adjacent to the multi-replicon bacteria *Rhodobacter* and *Burkholderia* are generally rich in plasmids or megaplasmids, while almost all species adjacent to the genus *Pseudoalteromonas* are single-replicon species without any plasmids (2). This may imply that chromids have diverse origins in different genera. *Pseudoalteromonas* is a genus of multi-replicon bacteria widely distributed in the marine environment, and it has been used as a model organism for studying adaptation mechanisms to cold environments (27). Species within this genus produce a rich variety of metabolites and play an important role in the ecological environment in which they are found (27–29). Among the species in this genus, the chromids exhibit different replication modes, including unidirectional and bidirectional replication (30). Considering the complex genome composition and biological importance of this genus, investigating the mutation features and evolutionary patterns of different replicons in *Pseudoalteromonas* will provide valuable insights into the species diversity and can also provide theoretical support for a deeper exploration of the origin and evolution of different types of replicons in bacteria and thus the relationships of their ecological niches.

In this study, we firstly used comparative genomics methods to analyze the sequence composition characteristics of chromosomes and chromids in *Pseudoalteromonas* species. Then we used the wild-type (WT) and MMR-deficient (Δ*mutS*) ancestral strains of two species, *P*. sp. LC0214 (with unidirectionally replicated chromids) and *P*. sp. JCM12884^T^ (with bidirectionally replicated chromids) to investigate the mutation features across different types of replicons at the whole-genome level of *Pseudoalteromonas* by mutation accumulation (MA) experiments combined with whole-genome-sequencing (MA-WGS) strategy. The similarities in sequence and evolutionary features between different replicons provide clues as to the evolutionary diversity of different replicons in the genus *Pseudoalteromonas*.

## RESULTS

### Sequence composition of chromosomes and chromids in *Pseudoalteromonas* species

To investigate the replicon sequence characteristics of the multi-replicon genus *Pseudoalteromonas*, 22 *Pseudoalteromonas* species fully assembled and annotated from the NCBI database were chosen to be reanalyzed (NCBI Genome data update date October 2023; Table S1). Among these, two species, *P. aliena* and *P. rhizosphaerae*, only had a primary replicon chromosome without chromid (Table S1). The chromosome sizes of the two single-replicon species were larger than those of the multi-replicon species (Table S1). GC skew analysis showed that the primary replicon chromosomes of the 22 species were symmetrical with respect to their replication origin and terminus (Fig. S1a). The secondary replicon chromids of two species *P*. sp. JCM12884^T^ and *P. piratica* were also symmetrical, indicating that the bidirectional replication was an adaptation of the chromids in the two species (Fig. S1b). In contrast, no apparent symmetrical phenomenon was observed in the chromids of the other 18 species, suggesting they were unidirectional (Fig. S1b). This confirms that species with unidirectional chromids are more prevalent within the genus *Pseudoalteromonas* (30).

**Fig 1:**
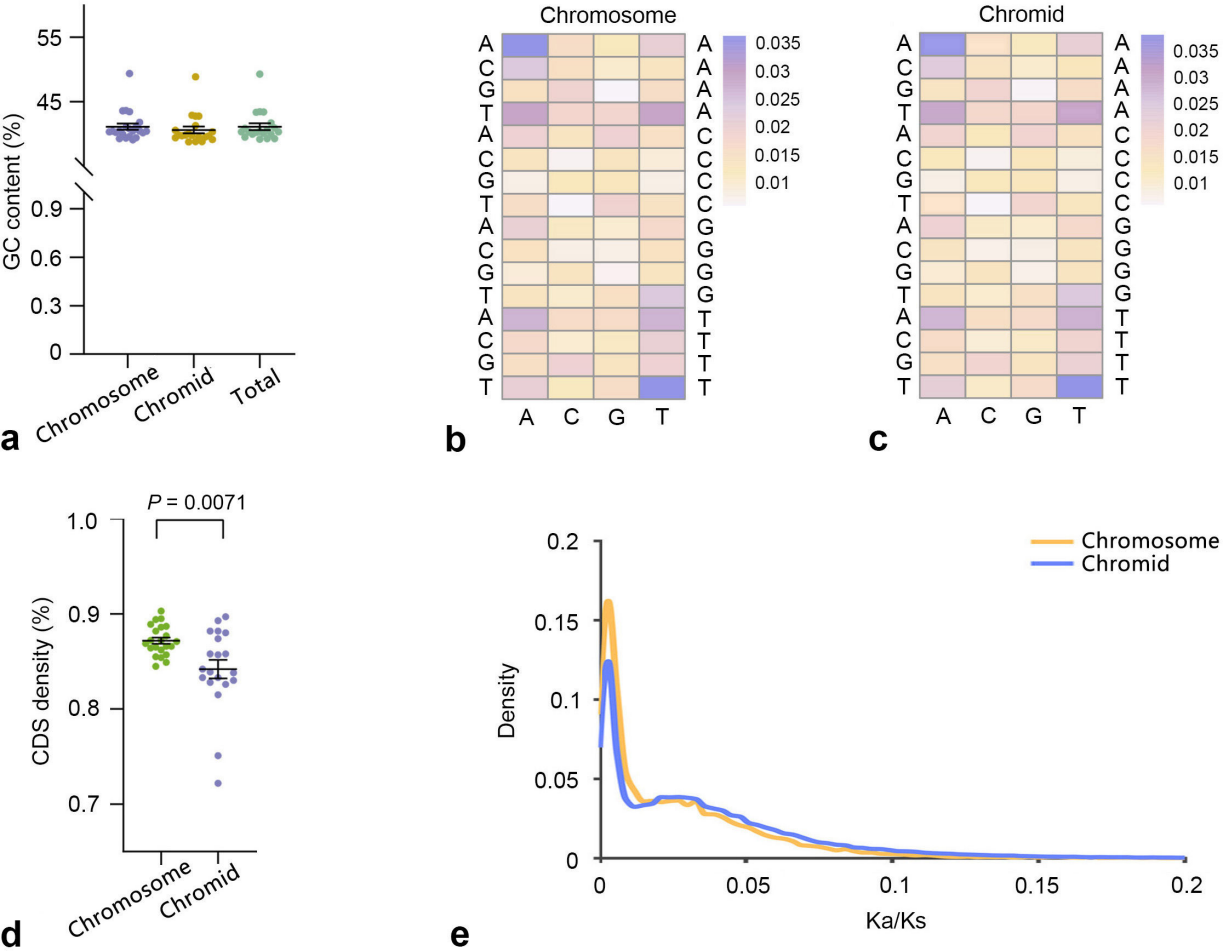
Sequence composition characteristics of chromosomes and chromids in 22 species of the genus *Pseudoalteromonas*. (a) GC content analysis (Mann-Whitney U test, *P* = 0.12). (b) Frequency of 64 trinucleotide compositions in the chromosome. (c) Frequency of 64 trinucleotide compositions in chromid. (d) CDS density (Mann-Whitney U test, *P* = 0.0071). (e) Ka/Ks values of single-copy orthologous genes. The orange curve represents the chromosome and the blue curve represents the chromid in unidirectional replication. The error bars denote SE.

Further comprehensive analyses identified similarities and differences in genomic sequence characteristics of the 22 species, including GC content, trinucleotide composition frequency, coding sequence (CDS) density, and Ka/Ks values of single-copy homologous genes of the chromosomes and chromids. There was no significant difference in GC content between the chromosomes and chromids of the 22 species, in which the difference between chromosomes and chromids was less than 1% (Mann-Whitney U test, *P* = 0.12; Fig. 1a). The ratios of the trinucleotides AAA and TTT were higher than other trinucleotide compositions in chromosomes, which was also observed in chromids (Fig. 1b and c). The CDS density in chromids was significantly lower than that in chromosomes (Mann-Whitney U test, *P* = 0.0071; Fig. 1d). The Ka/Ks values of single-copy orthologous genes of chromosomes and chromids were generally in the range of 0 to 0.1, indicating that the single-copy orthologous genes in both chromosomes and chromids were under strong negative selection (Fig. 1e).

### Phylogenetic tree construction and gene synteny analyses in the genus *Pseudoalteromonas*

**Fig 2:**
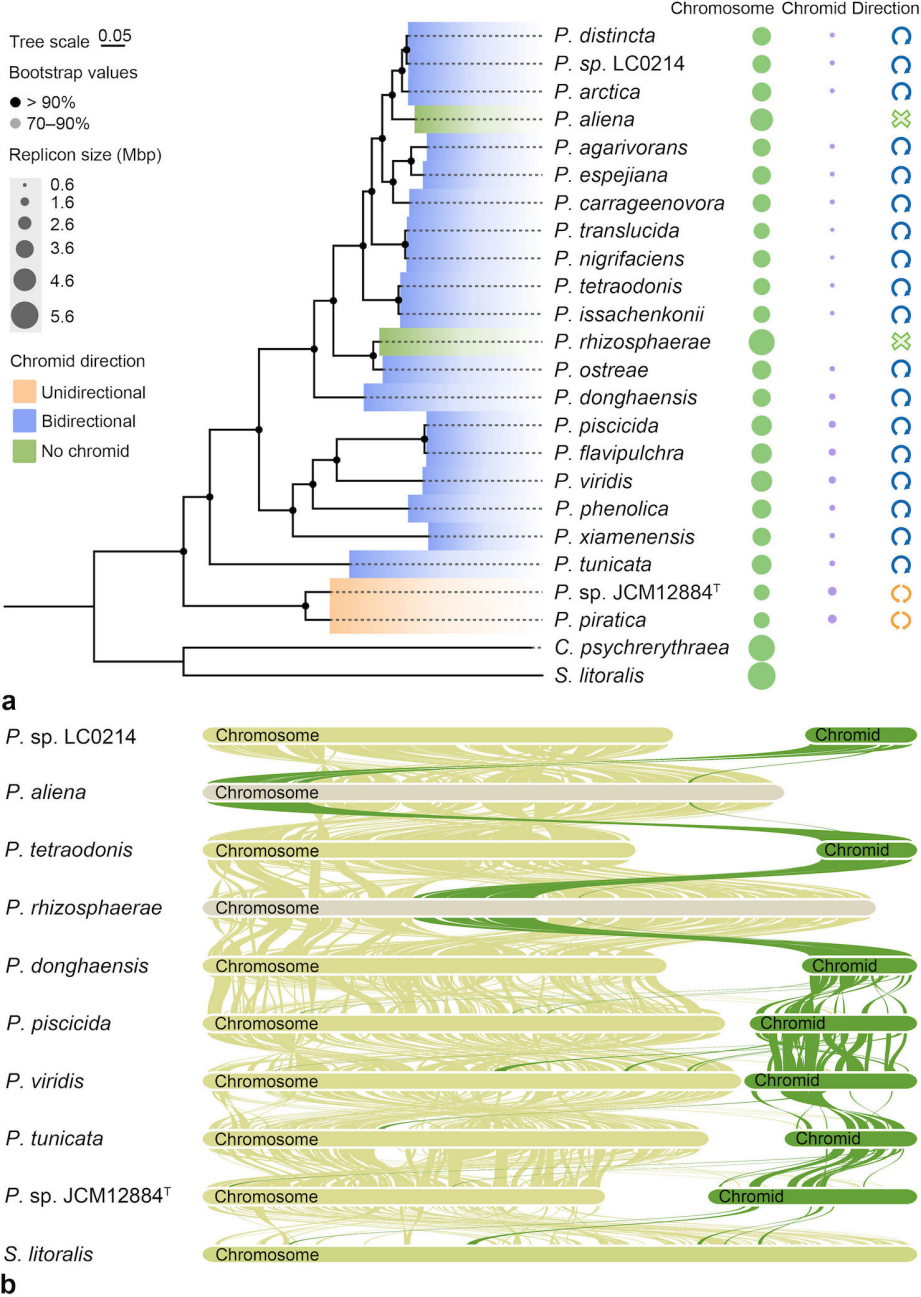
Species phylogenetic tree of the *Pseudoalteromonas* genus constructed using the maximum likelihood method and gene synteny analysis with MCScanX. (a) *Pseudoalteromonas* species phylogenetic tree constructed with the amino acid sequences of single-copy orthologous genes. The best model is LG+F+R6 as determined by AIC and BIC tests. The Bootstrap value is greater than 90%. Green and purple dots represent the size of the chromosome and chromid, respectively. The blue circle indicates unidirectional replication of the chromid, the orange circle indicates bidirectional replication of the chromid, and the green cross symbol indicates that the species does not contain a chromid. (b) Gene synteny analysis of selected *Pseudoalteromonas* species.

Although there were similarities shown in sequence composition characteristics between chromosomes and chromids within *Pseudoalteromonas* species, more details in the evolutionary patterns were necessary to be uncovered. We constructed the species phylogenetic tree and analyzed the gene synteny of chromosomes and chromids within several selected species on different evolutionary branches. The phylogenetic tree showed that the two species with bidirectionally replicated chromids clustered on one evolutionary branch, while the two single-replicon species clustered on a branch within the species with unidirectionally replicated chromids (Fig. 2a). Additionally, the phylogenetic topology in evolutionary branches of chromosomes and chromids in the corresponding species was almost identical (Fig. S2). There was also indicated a trend where the sequence size of chromids increased from the unidirectionally replicated to the bidirectionally replicated chromids (Table S1; Fig. 2a). These findings suggested that the diverse differentiation of chromids may have influenced the structure of evolutionary branches.

**Fig 3:**
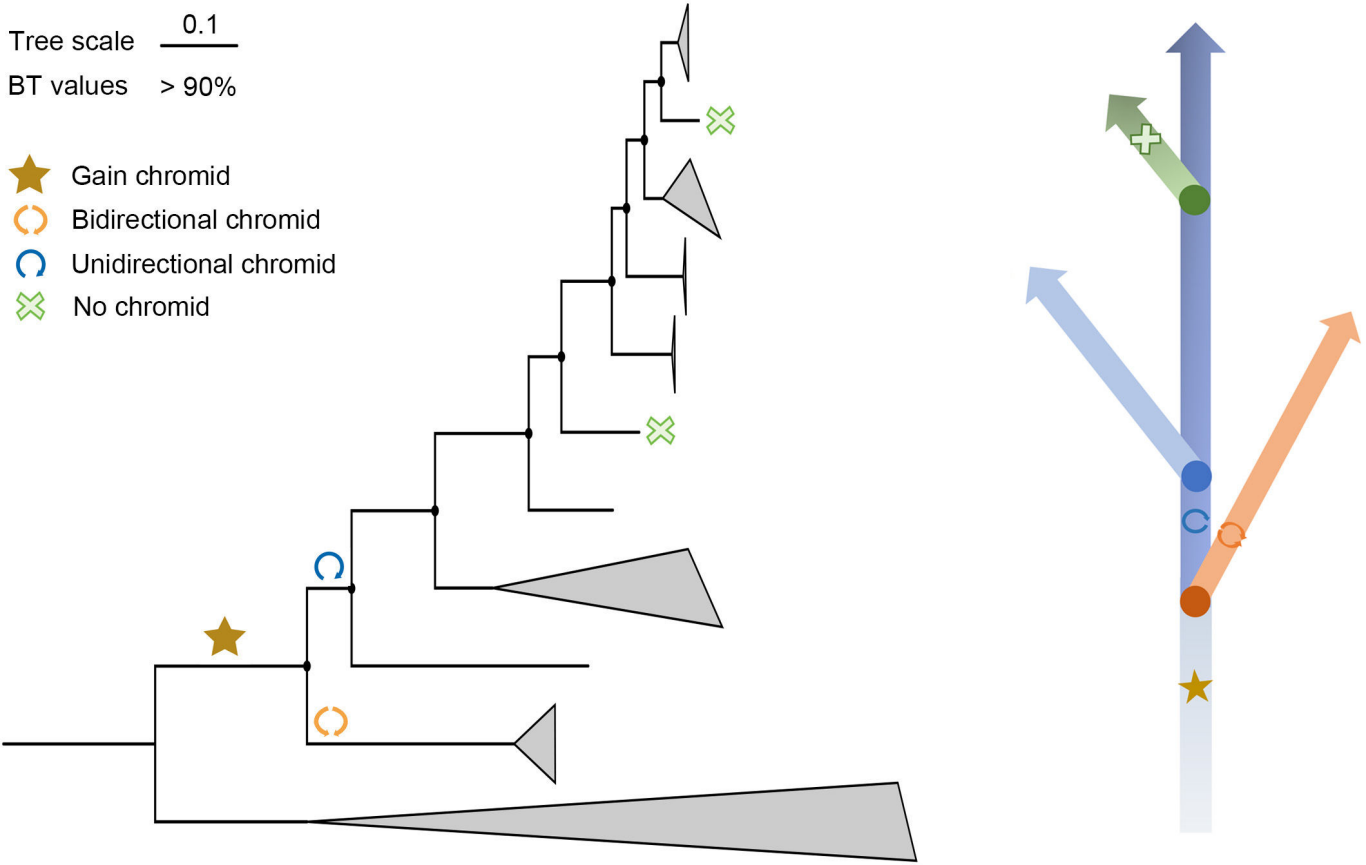
Evolutionary model diagram of species in the *Pseudoalteromonas* genus. The symbol “star” represents the acquisition of chromid, the blue circle indicates unidirectional replication of the chromid, the orange circle indicates bidirectional replication of the chromid, and the green cross symbol indicates that the species does not contain a chromid.

We also observed that the syntenic regions of chromids increased gradually from bidirectionally replicated chromids to unidirectionally replicated chromids (Fig. 2b). Both the chromosome and chromid of *P*. sp. JCM12884^T^ had collinear regions with the chromosome of the outgroup single-replicon species *Saccharobesus litoralis* (Fig. 2b). Furthermore, each of the two single-replicon species, *P. rhizosphaerae* and *P. aliena*, had a region in its chromosome that was strongly syntenic with the chromids of adjacent species, while showing almost no synteny with the chromosomes of adjacent species (Fig. 2b). These observations suggest that the absence of chromids in *P. rhizosphaerae* and *P. aliena* may be due to the reintegration of the chromid into the chromosome in the ancestors of these species. Based on the above results, we constructed an evolutionary model of *Pseudoalteromonas* species (Fig. 3), and it could be speculated that gene flow or recombination events have occurred frequently between chromosomes and chromids, contributing to the similarities of different replicons in the genus *Pseudoalteromonas*.

### Spontaneous mutation rates and spectra at the whole-genome level in wild-type mutation accumulation lines of *P*. sp. LC0214 and *P*. sp. JCM12884^T^

Although the above comparative genomic analysis showed great similarities of sequence composition characteristics between chromosomes and chromids in *Pseudoalteromonas*, whether the mutational patterns of different replicons in the genus *Pseudoalteromonas* align with these findings remained to be examined. Previous studies have reported that different types of replicons in multi-replicon species experience unique evolutionary rates (11, 21–26). To uncover patterns of spontaneous mutation in different replicons within *Pseudoalteromonas* species, we selected two representative species, *P*. sp. LC0214 and *P*. sp. JCM12884^T^, for further studies on the spontaneous mutation features at the genomic level. *P*. sp. LC0214 has a unidirectionally replicated chromid (separated and assembled by our laboratory), whereas *P*. sp. JCM12884^T^ has been reported to have a bidirectionally replicated chromid (30). We first verified the directionality of replicons by GC skew and deep sequencing of the genomic DNA coverage in both exponential and stationary growth stages of the two species (Fig. S3). We then conducted mutation accumulation experiments combined with whole-genome sequencing (WGS) using the WT ancestral strains of the two species (Tables S2 to S7).

**Fig 4:**
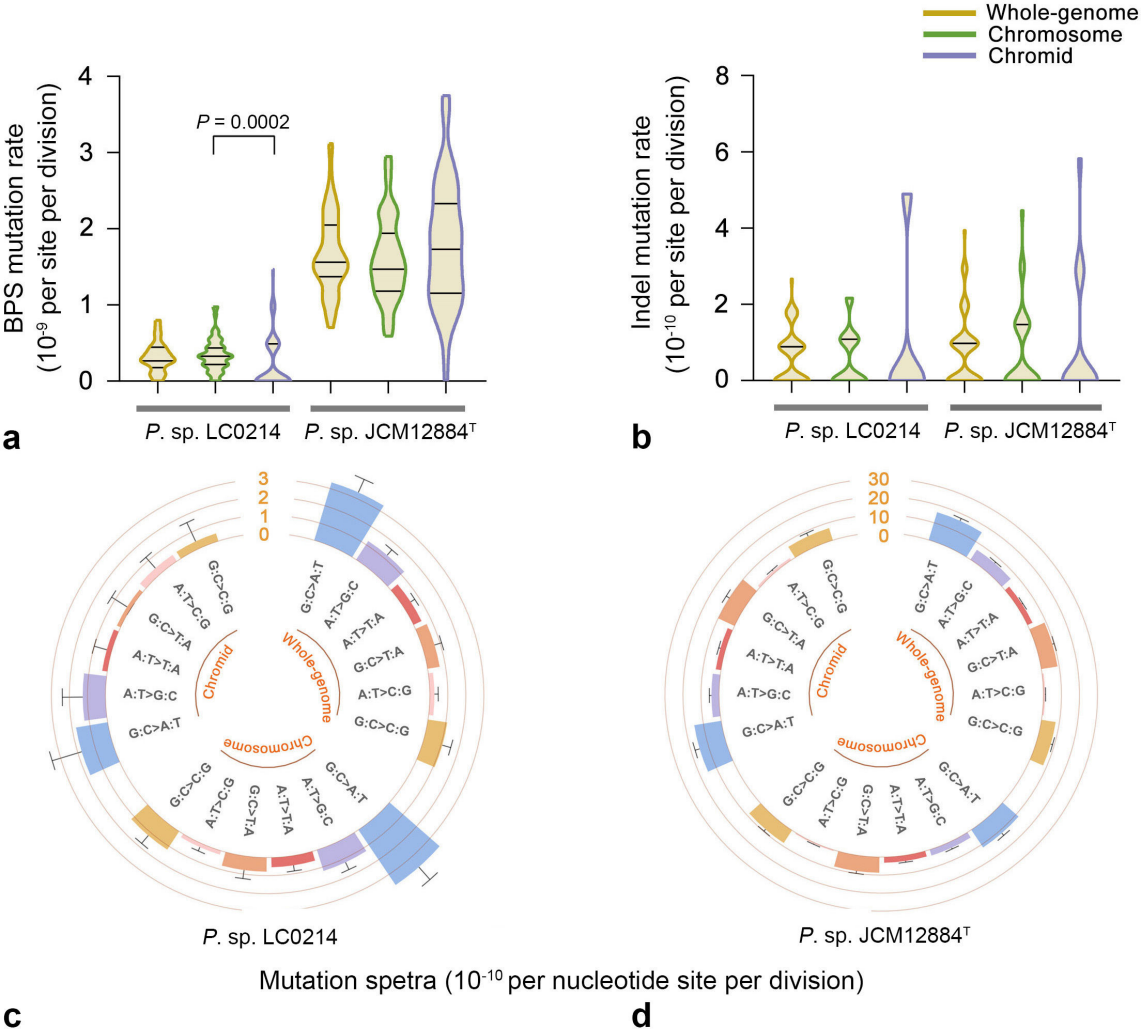
Mutation rates and spectra of *P*. sp. LC0214 and *P*. sp. JCM12884^T^ wild-type MA lines. (a) BPS mutation rates. (b) Indel mutation rates. (c and d) Mutation spectra of *P*. sp. LC0214 and *P*. sp. JCM12884^T^, respectively. All mutation rates are in units of per nucleotide site per cell division; error bars denote SE (a and b) and 95% confidence intervals (c and d).

In the *P*. sp. LC0214 WT MA lines, a total of 205 base-pair substitutions (BPSs) were detected, with an average whole-genome BPS mutation rate of 3.20 × 10^−10^ per nucleotide per cell division (95% confidence interval: 2.78 × 10^−10^, 3.67 × 10^−10^). Of these, 180 BPSs were located on the chromosome, and 25 BPSs were located on the chromid. The average BPS mutation rates of the chromosome and chromid were 3.43 × 10^−10^ (95% confidence interval: 2.95 × 10^−10^, 3.97 × 10^−10^) and 2.15 × 10^−10^ (95% confidence interval: 1.39 × 10^−10^, 3.17 × 10^−10^) per nucleotide per cell division, respectively. The BPS mutation rate of chromids was significantly lower than that of chromosomes (Mann-Whitney U test, *P* = 0.0002; Table 1; Fig. 4a). We detected 41 indels, with an average whole-genome indel mutation rate of 6.40 × 10^−11^ per nucleotide per cell division (95% confidence interval: 4.59 × 10^−11^, 8.68 × 10^−11^). Thirty indels were located on the chromosome and 11 indels were located on the chromid. The average indel mutation rates of the chromosome and chromid were 5.72 × 10^−11^ (95% confidence interval: 3.86 × 10^−11^, 8.17 × 10^−11^) and 9.46 × 10^−11^ (95% confidence interval: 4.72 × 10^−11^, 1.69 × 10^−10^) per nucleotide per cell division, respectively. There was no significant difference in the indel mutation rate between the chromid and chromosome levels (Mann-Whitney U test, *P* = 0.10; Table 1; Fig. 4b). Mutation spectrum analysis showed that transition mutations and transversion mutations G:C→C:G were the main mutation types in chromosomes, while transitions were the main mutation type in chromids (Fig. 4c).

**TABLE 1:** Mutation information^*a*^

Species	Strain	Level	BPSs	μ_BPS_			ts/tv	AT bias	Indels	μ_Indel_			Ins/Del
				Average	CID	CIU				Average	CID	CIU	
*P*. sp. LC0214	WT	Whole-genome	205	3.20	2.78	3.67	1.81	2.71	41	0.64	0.46	0.87	0.21
		Chromosome	180	3.43	2.95	3.97	1.77	3.10	30	0.57	0.39	0.82	0.25
		Chromid	25	2.15	1.39	3.17	2.13	1.19	11	0.95	0.47	1.69	0.1
*P*. sp. LC0214	Δ*mutS*	Whole-genome	29,738	693.68	685.82	701.61	128.30	0.74	942	21.97	20.61	23.45	0.72
		Chromosome	24,421	696.17	687.47	704.96	131.72	0.73	763	21.8	20.23	23.35	0.75
		Chromid	5,317	682.44	664.22	701.04	114.59	0.80	179	23	19.73	26.60	0.60
*P*. sp. JCM12884^T^	WT	Whole-genome	1,147	16.76	15.80	17.76	0.80	4.80	54	0.79	0.59	1.03	0.23
		Chromosome	729	16.10	14.95	17.31	0.74	5.03	34	0.75	0.52	1.05	0.26
		Chromid	418	18.05	16.36	19.87	0.92	4.48	20	0.86	0.53	1.33	0.18
*P*. sp. JCM12884^T^	Δ*mutS*	Whole-genome	16,787	303.23	298.66	307.85	30.09	1.19	401	7.24	6.55	7.99	0.60
		Chromosome	11,260	307.50	301.85	313.23	31.83	1.21	245	6.69	5.90	7.61	0.62
		Chromid	5,527	294.89	287.17	302.77	27.06	1.15	156	8.32	7.07	9.74	0.58

^*a*^ BPSs, total number of base-pair substitutions in the MA lines; μ_BPS_, base-pair substitution mutation rate; ts/tv, the ratio of transitions/transversions; AT bias, mutation bias in the A/T direction; indels, number of insertions and deletions; μ_Indel_, indel mutation rate; Ins/Del, the ratio of insertions/deletions. All mutation rates are in units of ×10^−10^ per nucleotide site per cell division, CID and CIU denote 95% Poisson confidence intervals (significant difference showed in BPS mutation rate between chromosome and chromid in *P*. sp. LC0214 wild-type MA lines, Mann-Whitney U test, *P* = 0.0002).

In the *P*. sp. JCM12884^T^ WT MA lines, a total of 1,147 BPSs were detected, with an average whole-genome BPS mutation rate of 1.68 × 10^−9^ per nucleotide per cell division (95% confidence interval: 1.58 × 10^−9^, 1.78 × 10^−9^). In this line, 729 BPSs were located on the chromosome, and 418 BPSs were located on the chromid. The average BPS mutation rates of the chromosome and chromid were 1.61 × 10^−9^ (95% confidence interval: 1.49 × 10^−9^, 1.73 × 10^−9^) and 1.81 × 10^−9^ (95% confidence interval: 1.64 × 10^−9^, 1.99 × 10^−9^) per nucleotide per cell division, respectively. There was no significant difference in BPS mutation rates between the chromosome and chromid levels (Mann-Whitney U test, *P* = 0.32; Table 1; Fig. 4a). We detected 54 indels, with an average whole-genome indel mutation rate of 7.89 × 10^−11^ per nucleotide per cell division (95% confidence interval: 5.93 × 10^−11^, 1.03 × 10^−10^). Thirty-four indels were located on the chromosome and 20 indels were located on the chromid. The average indel mutation rates of the chromosome and chromid were 7.51 × 10^−11^ (95% confidence interval: 5.20 × 10^−11^, 1.05 × 10^−10^) and 8.64 × 10^−11^ (95% confidence interval: 5.28 × 10^−11^, 1.33 × 10^−10^) per nucleotide per cell division, respectively. There was no significant difference in the indel mutation rates between the chromosome and chromid levels (Mann-Whitney U test, *P* = 0.31; Table 1; Fig. 4b). Mutation spectrum analysis showed that transitions G:C→A:T and transversions G:C→T:A and G:C→C:G were the main mutation types at both the chromosome and chromid levels (Fig. 4d).

At the whole-genome scale, we uncovered the spontaneous mutation rates and mutation spectra of chromosomes and chromids in wild-type MA lines of *P*. sp. LC0214 and *P*. sp. JCM12884^T^. There was nearly a five times difference in BPS mutation rate between the two wild-type strains. The differences might be explained by distinct evolutionary pressures involved in different habitats between the two species (27, 31). Within species, there were no significant differences in mutation rates and mutation spectra between chromosomes and chromids in *P*. sp. JCM12884^T^, while in *P*. sp. LC0214, compared to chromids, chromosomes have a 1.5-fold higher mutation rate, showing a certain degree of mutational variation. This variation is also reflected in the mutation spectra of the two replicons. Within multi-replicon species involved in different genera, the evolutionary trajectories and levels of secondary replicons are not consistent, implying that there are various possibilities for the evolutionary divergence between chromosomes and chromids within different species (2). Additionally, the origins of secondary replicons are varied, which may also cause the difference in mutation rates between replicons within multi-replicon species (2, 32). However, it is difficult to conclusively attribute these differences to the evolutionary features of replicons, as there were only 25 BPSs accumulated of the chromid in *P*. sp. LC0214 wild-type MA lines. This limited number of BPSs may have introduced statistical bias in the mutation rate analysis between replicons.

### Spontaneous mutation rates and spectra at the whole-genome level in Δ*mutS* MA lines of *P*. sp. LC0214 and *P*. sp. JCM12884^T^

The low mutation rates observed in the two wild-type species suggested that the vast majority of mismatches generated during replication were repaired by DNA mismatch repair systems (MMR). To investigate pre-mutation patterns, we constructed MMR-deficient (Δ*mutS*) strains of *P*. sp. LC0214 and *P*. sp. JCM12884^T^ (Fig. S4) and employed the MA-WGS strategy using the two Δ*mutS* ancestral strains (Tables S2 to S7).

**Fig 5:**
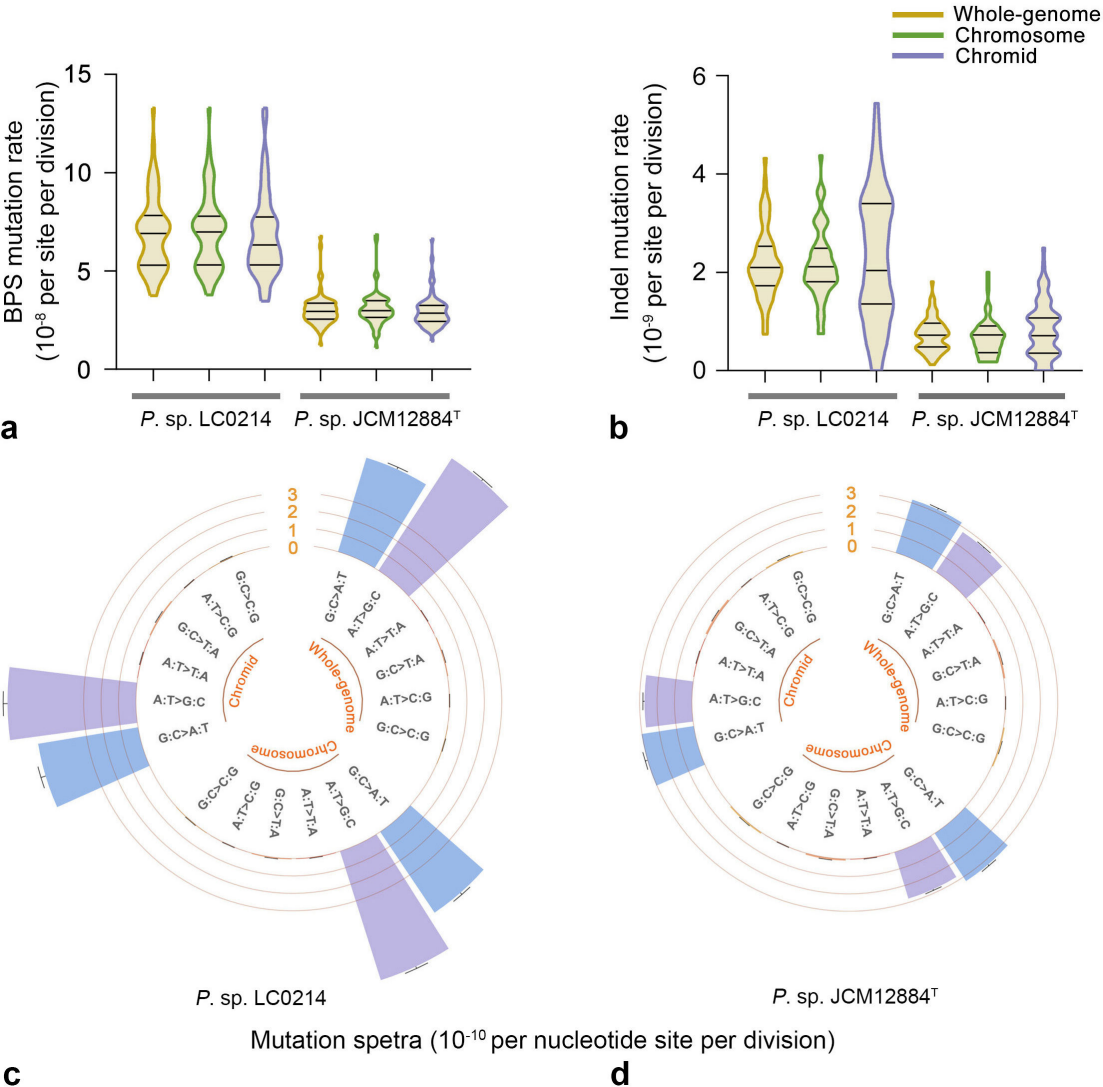
Mutation rates and spectra of *P*. sp. LC0214 and *P*. sp. JCM12884^T^ Δ*mutS* MA lines. (a) BPS mutation rate. (b) Indel mutation rate. (c and d) Mutation spectra of *P*. sp. LC0214 and *P*. sp. JCM12884^T^, respectively. All mutation rates are in units of per nucleotide site per cell division; error bars denote SE (a and b) and 95% confidence intervals (c and d).

In the *P*. sp. LC0214 Δ*mutS* MA lines, a total of 29,738 BPSs were detected, with an average whole-genome BPS mutation rate of 6.94 × 10^−8^ per nucleotide per cell division (95% confidence interval: 6.86 × 10^−8^, 7.02 × 10^−8^). Of these, 24,421 BPSs were located on the chromosome and 5,317 BPSs were located on the chromid. The average BPS mutation rates of the chromosome and chromid were 6.96 × 10^−8^ (95% confidence interval: 6.87 × 10^−8^, 7.05 × 10^−8^) and 6.82 × 1 0^−8^ (95% confidence interval: 6.64 × 10^−8^, 7.01 × 10^−8^) per nucleotide per cell division, respectively (Table 1; Fig. 5a). We detected 942 indels, with an average whole-genome indel mutation rate of 2.20 × 10^−9^ per nucleotide per cell division (95% confidence interval: 2.06 × 10^−9^, 2.34 × 10^−9^). Of the indels, 763 were located on the chromosome, and 179 indels were located on the chromid. The average indel mutation rates of the chromosome and chromid were 2.18 × 10^−9^ (95% confidence interval: 2.02 × 10^−9^, 2.34 × 10^−9^) and 2.30 × 10^−9^ (95% confidence interval: 1.97 × 10^−9^, 2.66 × 10^−9^) per nucleotide per cell division, respectively (Table 1; Fig. 5b). There was no significant difference in either BPS or indel mutation rate between the chromosome and chromid (BPS: Mann-Whitney U test, *P* = 0.62; indel: Mann-Whitney U test, *P* = 0.79). Mutation spectrum analysis showed that the main mutation types were transitions at both the chromosome and chromid levels (Table 1; Fig. 5c).

In the *P*. sp. JCM12884^T^ Δ*mutS* MA lines, a total of 16,787 BPSs were detected, with an average whole-genome BPS mutation rate of 3.03 × 10^−8^ per nucleotide per cell division (95% confidence interval: 2.99 × 10^−8^, 3.08 × 10^−8^). Of these, 11,260 BPSs were located on the chromosome, and 5,527 BPSs were located on the chromid. The average BPS mutation rates at the chromosome and chromid levels were 3.07 × 10^−8^ (95% confidence interval: 3.02 × 10^−8^, 3.13 × 10^−8^) and 2.95 × 10^−8^ (95% confidence interval: 2.87 × 10^−8^, 3.03 × 10^−8^) per nucleotide per cell division, respectively (Table 1; Fig. 5a). We detected 401 indels, with an average whole-genome indel mutation rate of 7.24 × 10^−10^ per nucleotide per cell division (95% confidence interval: 6.55 × 10^−10^; 7.99 × 10^−10^). Of the indels, 245 were located on the chromosome, and 156 indels were located on the chromid. The average indel mutation rates at the chromosome and chromid levels were 6.69 × 10^−10^ (95% confidence interval: 5.90 × 10^−10^, 7.61 × 10^−10^) and 8.32 × 10^−10^ (95% confidence interval: 7.07 × 10^−10^, 9.74 × 10^−10^) per nucleotide per cell division, respectively (Table 1; Fig. 5b). There were no significant differences in either BPS or indel mutation rate between the chromosome and chromid (BPS: Mann-Whitney U test, *P* = 0.15; indel: Mann-Whitney U test, *P* = 0.42). Mutation spectrum analysis showed that the main mutation types were transitions at both the chromosome and chromid levels (Table 1; Fig. 5d).

In both *P*. sp. LC0214 and *P*. sp. JCM12884^T^ Δ*mutS* MA lines, no significant differences were observed in BPS/indel rates or mutation spectra between chromosomes and chromids. This indicates that whether the chromid replicated unidirectionally or bidirectionally, the spontaneous mutation features during replication were highly similar to those of chromosomes within species. However, the 95% confidence intervals for BPS and indel mutation rates between the Δ*mutS P*. sp. LC0214 and *P*. sp. JCM12884^T^ lines do not overlap, indicating a significant difference in mutation rates between the two MMR-deficient strains. In addition, our results revealed that MMR dysfunction led to an exponential increase in the genomic mutation rates in both *P*. sp. LC0214 and *P*. sp. JCM12884^T^, indicating the extremely high repair efficiency of functional MMR for post-replication mismatches. The BPSs obtained in Δ*mutS* MA lines were sufficient for further detailed analysis of mutation characteristics (Table 1).

### BPS context-dependent rates and fluctuation variations of Δ*mutS* MA lines of *P*. sp. LC0214 and *P*. sp. JCM12884^T^

**Fig 6:**
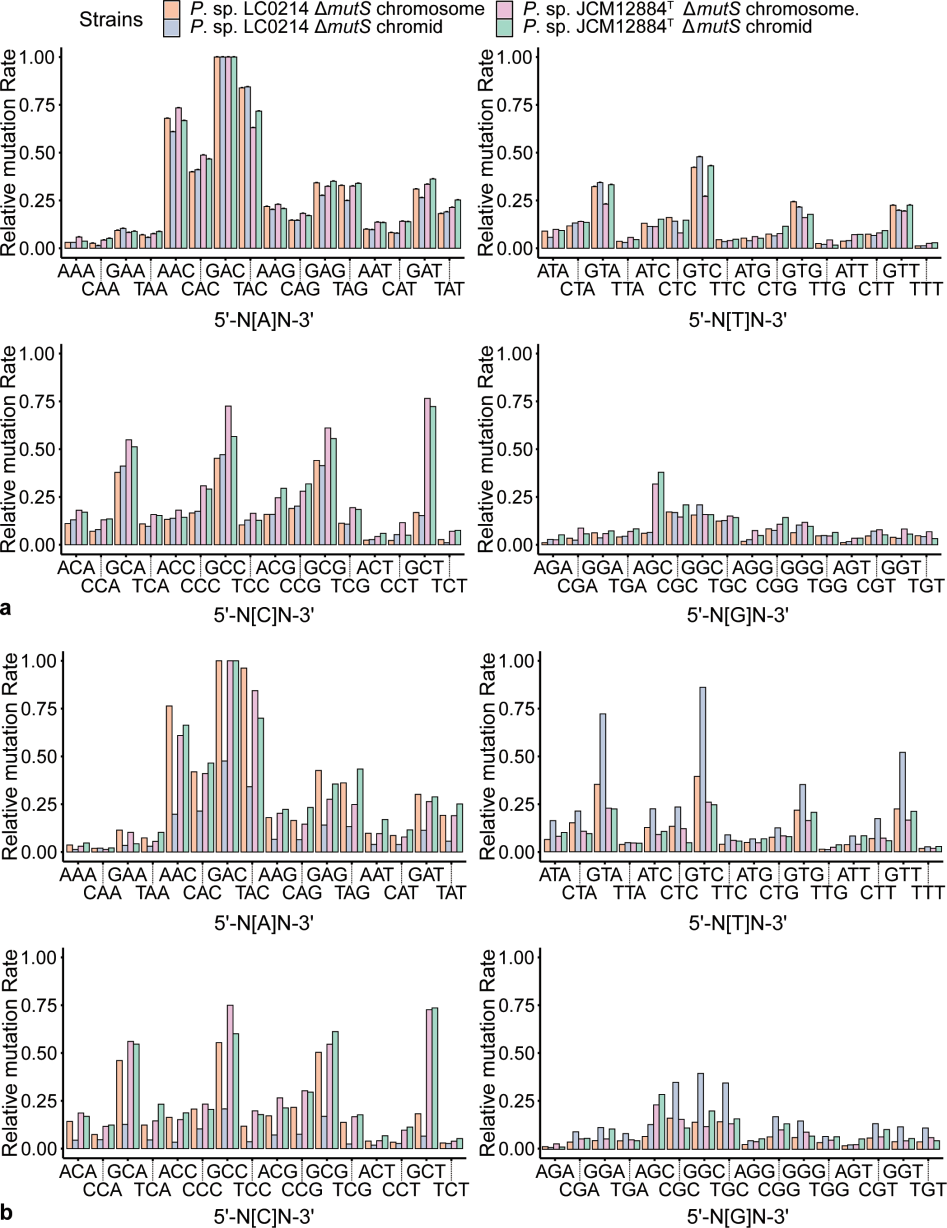
Relative context-dependent mutation rates of *P*. sp. LC0214 and *P*. sp. JCM12884^T^ Δ*mutS* MA lines. (a) Leading strand of chromosomes and chromids. (b) Lagging strand of chromosomes and chromids. The normalized mutation rates for the leading and lagging strands of the four replicons were calculated using the data provided in the supplemental file (S7). Each data set of 64 rates was normalized by dividing all 64 rates by the largest rate in that data set.

Although chromids and chromosomes have nearly identical mutation rates and mutation spectra, we observed some differences in terms of BPS context-dependent rates and variations at the replicon level. In both *P*. sp. LC0214 and *P*. sp. JCM12884^T^ Δ*mutS* MA lines, the context-dependent mutation rates of nucleotides flanked by G/C were higher compared to those lacking G/C flanking (Table S7; Fig. S5). However, the 64 triplets’ BPS context-dependent rates in chromosomes and chromids showed differences in *P*. sp. LC0214 Δ*mutS* MA lines, but similarity in both the chromosome and chromid in the *P*. sp. JCM12884^T^ Δ*mutS* MA lines (Fig. S5). Considering the different replication models between unidirectional and bidirectional chromids, we calculated the relative 64 triplets’ BPS context-dependent rates in leading and lagging strands of each replicon, respectively. Generally, the 64 triplets’ BPS context-dependent rates were similar between the leading and lagging strands in the chromosome and chromid of *P*. sp. JCM12884^T^ Δ*mutS* MA lines (Fig. 6a and b). However, in *P*. sp. LC0214 Δ*mutS* MA lines, the 64 triplets’ BPS context-dependent rates showed greater differences between the leading and lagging strands in the chromosome and chromid, especially in the 5′-N[A/C]N-3′ triplets (Fig. 6b). It has been reported that replication via the rolling-circle mechanism is inherently unidirectional, and it is considered to be an asymmetric process because synthesis of the leading strand and synthesis of the lagging strand are uncoupled (33). This supports our speculation that the lagging strand of unidirectional chromid was exposed in single-stranded mode for a longer period during replication, potentially leading to specific mutations.

**Fig 7:**
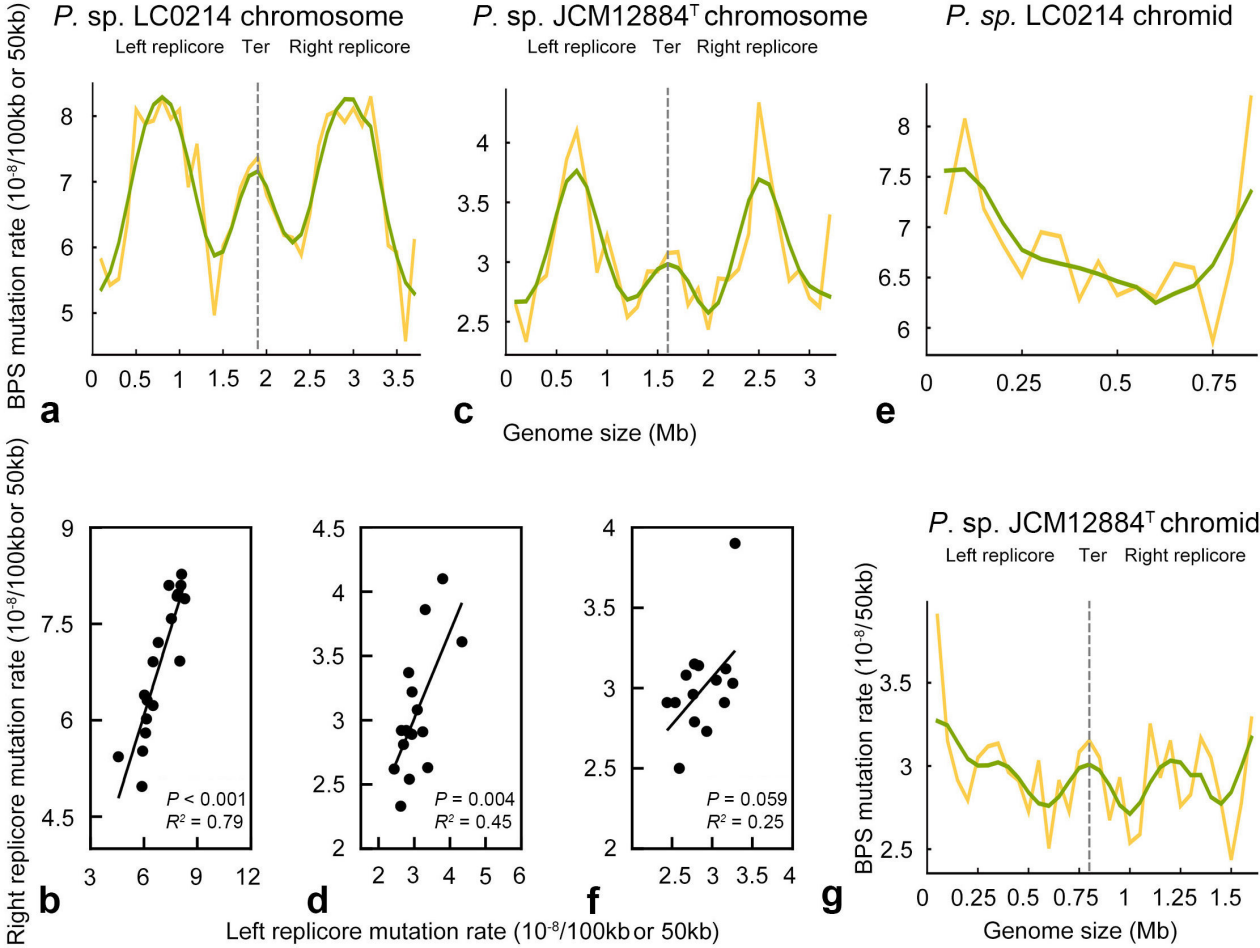
Patterns of BPS mutation rates of *P*. sp. LC0214 and *P*. sp. JCM12884^T^ Δ*mutS* MA lines in 100 kb (chromosome) and 50 kb (chromid) intervals extending clockwise from the origin of replication (oriC). (a), (c), (e), and (g) represent the BPS mutation rate pattern of each replicon, respectively. The orange curve represents the BPS mutation rate in each bin (for chromosome, per 100 kb; for chromid, per 50 kb), and the green curve represents the BPS mutation rate patterns transformed by Daubechies Wavelet. (b), (d), and (f) represent the results of correlation analysis of the BPS mutation rate of the left and right replicores in chromosome and chromid.

Previous studies have reported that the BPS mutation rates present a symmetrical wave-like distribution at genome-wide level, which was discovered by Foster et al. in MMR-deficient strain of *Escherichia coli* (34). To reveal the mutation patterns at the replicon level in the *P*. sp. LC0214 and *P*. sp. JCM12884^T^ Δ*mutS* MA lines, we divided the chromosomes into 100 kb intervals (starting from the origin of replication) to calculate the BPS mutation rate in each interval for further examining the variation pattern in the BPS mutation rate at the chromosome level. Due to the shorter length of chromids compared to chromosomes, we divided each chromid into 50 kb intervals (starting from the origin of replication) to calculate the BPS mutation rate in each interval. The BPS mutation rates along chromosomes exhibited a symmetrical wave-like pattern, with lower mutation rates near the origin of replication and higher rates in the middle and terminal regions (*P*. sp. LC0214: *R²* = 0.79, *P* < 0.0001; *P*. sp. JCM12884^T^: *R²* = 0.45, *P* = 0.0043; Fig. 7a through d). It was proposed that this symmetrical pattern was closely related to genomic features, specifically predicting the highest mutation density in regions with high supercoiling (34). Deeper investigations were conducted to find underlying causes of this wave-like symmetrical distribution in *E. coli*, and the collective evidence from the research suggests that a single factor cannot fully account for this phenomenon (35).

In chromids, it showed that the BPS mutation rate was higher at the replication origin (or the replication terminus) without the symmetrical wave-like pattern observed across the entire chromid in *P*. sp. LC0214 Δ*mutS* MA lines (Fig. 7e). Although the higher BPS mutation rate near the replication origin region (oriC) of the chromid in *P*. sp. JCM12884^T^ Δ*mutS* MA lines, a symmetrical wave-like pattern of mutation rates was still observed (*R²* = 0.25, *P* = 0.059; Fig. 7f and g). It has been reported that the replication of chromids initiates after that of the chromosome, and it replicates only once per cell cycle (36, 37). The delayed initiation of chromid replication after chromosome replication initiation was also observed in *Pseudoateromonas* (30). We speculate that the higher BPS mutation rate observed near the oriC region of the chromid in *P*. sp. JCM12884^T^ might be attributed to differences in the timing of replication initiation between different replicons.

Considering the above findings and the different replication direction of the chromids in *P*. sp. LC0214 and *P*. sp. JCM12884^T^, it can be inferred that the difference in the BPS variation patterns between chromosomes and chromids was associated with the replication direction. Based on these results, we suggest that the mutation features of bidirectional replicated chromids are more similar to those of chromosomes than those of unidirectional replicated chromids within *Pseudoalteromonas* species.

## DISCUSSION

In this study, we employed comparative genomics and MA-WGS strategies to investigate the sequence composition and spontaneous mutation features of primary replicons (chromosomes) and secondary replicons (chromids) in the multi-replicon genus *Pseudoalteromonas*. Similarities between chromosomes and chromids were observed in sequence trinucleotide frequency, GC content, and the Ka/Ks values of single-copy homologous genes. Gene synteny analysis revealed higher synteny between smaller chromids compared to larger chromids within *Pseudoalteromonas*. Notably, two single-replicon species within this genus exhibited significant syntenic regions with chromids of neighboring species, whereas these syntenic regions on single-replicon species chromosomes were not syntenic with the chromosomes of neighboring species. MA-WGS analyses showed that the mutation rates and mutation spectra of chromosomes and chromids within MMR-deficient strains *P*. sp. LC0214 and *P*. sp. JCM12884^T^ were highly similar, respectively. Despite the high similarity between chromosomes and chromids in both sequence composition features and mutation rates, more differences were found in the BPS context-dependent mutation rates and BPS mutation variation pattern between the unidirectionally replicated chromid and chromosome of *P*. sp. LC0214, while fewer differences were shown between the bidirectionally replicated chromid and chromosome of *P*. sp. JCM12884^T^.

Although there were some noticeable differences in mutation rates and mutation spectra between the two species, *P*. sp. LC0214 and *P*. sp. JCM12884^T^, this was understandable as the two species had different living environments. It means that during the evolution of species, they may be under the evolutionary pressures of different sources and types. *Pseudoalteromonas* is ubiquitous in marine ecosystems, with members occupying a wide range of habitats (27, 31). *P*. sp. JCM12884^T^ was isolated from the surface of the sponge *Mycale adhaerens* (38), and *P*. sp. LC0214 was isolated from sea lettuce (39). The mutational differences might be explained by distinct evolutionary pressures involved in different habitats between the two species (27, 31). Similar differences in mutation rates and spectra have also been observed between *Vibrio cholerae* and *V. fischeri* within the marine bacterial genus *Vibrio* (32), suggesting that variations in spontaneous mutation features among different species in *Pseudoalteromonas* are explicable. Besides the above, when the two species experience different levels of oxidative damage and have varying DNA repair efficiencies, these disparities could be reflected in differences in mutation rates and spectra.

In multi-replicon bacteria, the genome primarily comprises a primary replicon (the chromosome) and secondary replicons (chromids) (2). The term “Chromid” was first defined by Harison et al. to distinguish it from chromosomes and plasmids (1). Two main hypotheses describe the formation of the essential secondary replicon: the schism hypothesis and the plasmid hypothesis (40–42). However, systematic studies on the evolutionary patterns of multi-replicon bacteria are lacking. With the rapid advancement and widespread adoption of high-throughput sequencing, a vast amount of genomic information has been made public for numerous species. As the rich data set provides a solid foundation for investigating the evolution across species, we constructed the phylogenetic tree based on single-copy orthologous genes of the 193 species before we started the research on *Pseudoalteromonas* (Table S8; Fig. S6). The phylogenetic tree showed that several *Rhizobium* species were cross-distributed on the evolutionary branches containing *Sinorhizobium* and *Agrobacterium* genera, which was consistent with the results of the bacterial phylogenetic tree constructed by diCenzo et al. (2) based on ribosomal RNA sequences. It has been reported that 25% to 30% of genes on the *S. meliloti* chromid have orthologs on the *A. tumefaciens* chromosome (43). Considering the large ecological and evolutionary differences between different genera, it appears to be overly simplistic to generalize the origin and formation pattern of chromids in multi-replicon genera (44).

Previous studies have reported that the presence of secondary replicons is associated with the expansion of the whole-genome size of the species (2, 45). Generally, the sizes of chromids were smaller than those of chromosomes, in which the size of unidirectionally replicated chromids were smaller than those of bidirectionally replicated chromids in the genus *Pseudoalteromonas* (Table S1). Moreover, the synteny of smaller chromids was generally higher than that of larger chromids among *Pseudoalteromonas* species (Fig. 1b). This may suggest that unidirectionally replicated chromids have expanded and evolved to bidirectional replication to reduce replication time (30). However, the phenomenon observed in the genus *Pseudoalteromonas* seemed to be inconsistent with this pattern, as the sizes of chromosomes of species with bidirectionally replicated chromids were smaller than those of species with unidirectionally replicated chromids (Table S1). In addition, the sizes of chromosomes of the two single-replicon species, *P. rhizosphaerae* and *P. aliena*, were larger than those of all other multi-replicon species within the genus *Pseudoalteromonas* (Table S1). The absence of chromids in these species might be attributed to the potential reintegration of the chromid into the chromosome in their ancestors. Furthermore, there were similarities observed in sequence composition and the gene synteny between the chromosomes and chromids of *Pseudoalteromonas* species. The two single-replicon species show strong synteny with the chromids of adjacent species, yet lack synteny with the chromosomes of adjacent species. According to the above results, we speculated that gene flow or recombination events may have occurred frequently between chromosomes and chromids, contributing to the similarities of different replicons in the genus *Pseudoalteromonas*.

It has been suggested that replicons in multi-replicon species could experience different evolutionary rates and pressures, in which the mutation rates of specific sequences located on chromosomes were lower than those of chromids, indicating less purifying selection on chromids (11, 21). However, focusing solely on the mutation features of specific sequences can be biased for an in-depth understanding of bacterial evolution. At the whole-genome level, among the evolutionary characteristics of multi-replicon species, only a few species within the genera *Vibrio*, *Deinococcus*, and *Burkholderia* have been studied, without comprehensive comparisons between different replicons (32, 46, 47). Genomic mutations are a crucial driver of bacterial evolution (48–55), and deeply studying the mutation features can enhance our understanding of bacterial evolution (56–58). For further comparison of the similarities and differences in evolutionary patterns of replicons, we selected *P*. sp. LC0214 (with unidirectionally replicated chromids) and *P*. sp. JCM12884^T^ (with bidirectionally replicated chromids) of the *Pseudoalteromonas* species to investigate the mutation features between chromosomes and chromids at the whole-genome level. The MA-WGS results demonstrate high similarity in BPS mutation rates, indel mutation rates, and mutation spectra between chromosomes and chromids, especially in the MMR-deficient MA lines. Combined with the comparative genomic analysis and MA-WGS results, we proposed that within the genus *Pseudoalteromonas*, chromosome fragments may have fused with intracellular plasmids, and these fused sequences eventually evolved into chromids, where the chromids have undergone different degrees of species-specific differentiation. However, further downstream experiments are needed to verify this hypothesis.

Despite the high similarity in sequence characteristics and mutation rates between chromosomes and chromids in *Pseudoalteromonas* species, differences were found in the BPS context-dependent mutation rates and mutation variation patterns. In the *P*. sp. LC0214 MMR-deficient MA lines, there were differences observed in the 64 triplets’ BPS context-dependent rates between leading and lagging strand in chromosome and chromid. In contrast, in *P*. sp. JCM12884^T^ MMR-deficient MA lines, the 64 triplets’ BPS context-dependent rates were highly similar between the leading and lagging strand of the chromosome and chromid. Moreover, in the MMR-deficient MA lines, the BPS mutation rates exhibited a symmetrical wave-like pattern along both the chromosome and chromid of *P*. sp. JCM12884^T^, while this pattern was only observed in the chromosome of *P*. sp. LC0214. These findings may imply that there was a trend of unidirectionally replicated chromid evolving into bidirectionally replicated chromid, and there were closer evolutionary relations between bidirectionally replicated chromids and chromosomes. Besides the above, the difference in the variation pattern of BPS mutation rates at the chromid level in the two species may be due to different replication directions and also related to plasmids with different replication patterns, although further deep exploration should be conducted to verify this hypothesis (33, 59–62).

In summary, we analyzed the genomic information of the latest species within the genus *Pseudoalteromonas* and performed a detailed comparative analysis of the sequence composition and whole-genome mutation features of chromosomes and chromids built upon previous studies on *Pseudoalteromonas* (30). This study enriches our knowledge of genome composition characteristics and evolutionary patterns of multi-replicant bacteria. In addition, the observed similarities and differences in mutation patterns between different replicons in *Pseudoalteromonas* may provide new research directions and a foundation for further in-depth research on the origin and evolution of secondary replicon in bacteria.

## MATERIALS AND METHODS

### Analyses of the genomic sequence characteristics of *Pseudoalteromonas* replicons

To analyze replicon sequence characteristics of *Pseudoalteromonas* species, we downloaded the whole-genome sequences of the 22 fully assembled and annotated *Pseudoalteromonas* species from the NCBI database (https://www.ncbi.nlm.nih.gov/datasets/taxonomy/53246/) (NCBI Genome data update date October 2023). Among the 22 species, 20 are multi-replicon and two are single-replicon species (Table S1).

We first counted the G/C base number of each chromosome and chromid (20 species contain chromids) within the 22 species and calculated the GC content by dividing the G/C base number by the total base number of the replicon. As the trinucleotide composition frequency of genomic sequences is one of the important features reflecting genomic evolution, we calculated the trinucleotide composition frequency of the chromosomes and chromids of the 22 species. We calculated the frequency of 64 trinucleotides from the first base of the replicon sequence with a self-written script, with a window size of 3 bp and a step size of 1 bp. Then we counted the number of CDSs in each chromosome and chromid and calculated the CDS density by dividing the number of CDSs by the total base number of the replicon. We also reanalyzed the GC skew (30, 63, 64) of each replicon of the 22 species and used Orifinder to predict the origin of replication (65).

Calculating nonsynonymous (Ka) and synonymous (Ks) substitution rates is of great significance in understanding evolutionary dynamics of protein-coding sequences across closely related and yet diverged species (66, 67). It is known that the Ka/Ks ratio indicates neutral mutation when Ka equals Ks, negative (purifying) selection when Ka is less than Ks, and positive (diversifying) selection when Ka exceeds Ks (66, 68). To compare the selection intensity of genes on chromosomes and chromids, ParaAT.pl and PAML software (69) were used to calculate the Ka/Ks ratio of 1,428 single-copy homologous genes on chromosomes (22 species) and 98 single-copy homologous genes on chromids (20 species), respectively. The single-copy homologous genes were obtained using Orthofinder software (v-2.5.4) (70).

### Phylogenetic tree construction and synteny analyses of the genus *Pseudoalteromonas*

To uncover the phylogenetic relationships within different species of the *Pseudoalteromonas* genus, we downloaded the whole-genome coding region amino acid sequences of the above 22 fully assembled and annotated *Pseudoalteromonas* species, along with the two single-replicon outgroup species, *Saccharobesus litoralis* and *Colwellia psychrerythraea*. Single-copy orthologous genes were identified using OrthoFinder (v-2.5.4) (70), and the amino acid sequences of single-copy orthologous genes were aligned using Mafft (v-7.475) software with default parameters (71). IQ-TREE (v-2.1.4) (72) was used to construct the phylogenetic tree based on the maximum likelihood principle after alignment, and the phylogenetic trees were visualized using the Chiplot online website (73). Additionally, we constructed phylogenetic trees based on the single-copy orthologous gene amino acid sequences of the primary replicon (chromosome) and the secondary replicon (chromid), respectively.

To reveal the gene collinearity relationship between different replicons, we selected several representative species from different evolutionary branches based on the phylogenetic tree. MCScanX (74) software was used to perform gene collinearity analysis. The selected species were as follows: the outgroup single-replicon species *Saccharobesus litoralis* and the *Pseudoalteromonas* species *P*. sp. JCM12884^T^, *P. tunicata*, *P. viridis*, *P. piscicida*, *P. donghaensis*, *P. rhizosphaerae*, *P. tetraodonis*, *P. aliena*, and *P*. sp. LC0214.

### Replication direction verification of chromids in *P*. sp. LC0214 and *P*. sp. JCM12884^T^

Different replicated directions of replicons could be related to the evolutionary trajectory of species. To further investigate the evolutionary characteristics of chromosomes and chromids with different replicated direction, we selected two *Pseudoalteromonas* species, *P*. sp. LC0214 (*P*. sp. LC0214 was also called *P*. sp. LC2018020214 [39], isolated and verified from marine sediment by our laboratory) and *P*. sp. JCM12884^T^ (isolated from the surface of the sponge *Mycale adhaerens* [38]), for further study of their genomic mutation features. The genome of *P*. sp. LC0214 is composed of a chromosome and an unidirectionally replicated chromid, while the genome of *P*. sp. JCM12884^T^ is composed of a chromosome and a bidirectionally replicated chromid. TBtools software (75) was used first to perform gene collinearity analysis on different replicons of these two species.

In order to verify the replication direction of each replicon in the two species, the cells of each species were cultured in marine LB broth at 25°C and harvested at the exponential phase (~4 h) and the stationary phase (24 to 40 h). Genomic DNA was extracted using the MasterPure Complete DNA and RNA Purification Kit according to the product instructions. A 300 bp insert size library was constructed for each sample and sequenced with an Illumina Novaseq6000 system with PE150 bp reads. After being filtered by Fastp (v-1.0) (76), the clean reads were mapped to replicons (NZ_CP066804.1 for chromosome and NZ_CP066805.1 for chromid in *P*. sp. LC0214; NZ_CP011039.1 for chromosome and NZ_CP011040.1 for chromid in *P*. sp. JCM12884^T^) using Burrows-Wheeler Aligner (v-0.7.17) (77). The coverage of every base pair was calculated using the mpileup subprogram in SAMtools (78). The coverage data were further grouped in bins of 1 kbp.

### Construction of *P*. sp. LC0214 and *P*. sp. JCM12884^T^ DNA mismatch repair system deficient strains

To investigate pre-mutation patterns, we constructed MMR-deficient strains (Δ*mutS*) of *P*. sp. LC0214 and *P*. sp. JCM12884^T^ using markerless deletion of the *mutS* gene with the principle of homologous recombination (79). The method introduced a fragment of upstream and downstream homology arms of the *mutS* gene into the strains, replacing the wild-type *mutS* gene sequence via homologous recombination, thereby generating MMR-deficient knockout strains.

### Mutation accumulation procedures

Mutation accumulation (MA) experiments using bacteria are performed by repeatedly passing large numbers of initially ancestral cells through single-cell bottlenecks. This procedure prevents natural selection from promoting or eliminating nearly all mutations, except for the small subset with extremely large effects (49). Combining MA with WGS provides an essentially unbiased, genome-wide view of the mutation rate and the full molecular spectrum of mutations, yielding accurate estimates of these features across a wide variety of prokaryotic and eukaryotic microbes (50–53). To explore the whole-genome mutational features of different types of replicons in *P*. sp. LC0214 and *P*. sp. JCM12884^T^, we performed MA-WGS on the four ancestral strains (*P*. sp. LC0214 WT/Δ*mutS*, *P*. sp. JCM12884^T^ WT/Δ*mutS*). In this MA procedure, 60 MA lines were initiated of *P*. sp. LC0214 WT and Δ*mutS* strains, and 70 MA lines were initiated of *P*. sp. JCM12884^T^ WT and Δ*mutS* strains, respectively. All 260 MA lines were cultured on marine LB agar (Solarbio, Cat. No. L8290) at 25°C, with each line being single-colony transferred daily.

The MA experiments lasted 80 to 100 days, during which each WT MA line was transferred for 100 times and each Δ*mutS* MA line was transferred for 80 times on average. In order to estimate the cell divisions (t) between transfers (Num) by the colony-forming units, we performed serial dilution every ~30 days, by randomly choosing and razor-cutting a single colony from each of the five lines for the WT and the Δ*mutS* MA lines, based on the formula:


$$t=log_{2}\left(Num\right)$$

### DNA extraction, library construction, and whole-genome sequencing

After the last transfer, we picked a single colony for each final MA line as well as the ancestral line for each strain and cultured them in marine LB broth (Solarbio, Cat. No. L8291) overnight at 25°C. We then extracted genomic DNA using the MasterPure Complete DNA and RNA Purification Kit (Lucigen, Cat No. MC85200). Short-read libraries of DNA that met the concentration and quality requirements were constructed using an optimized protocol for the TruePrep DNA Library Prep Kit V2 for Illumina (Vazyme, Cat. No. TD501-01) and the TruePrep Index Kit V3 for Illumina (Vazyme, Cat. No. TD203). After performing agarose gel electrophoresis and cutting the target bands for recycling with the E.Z.N.A. Gel Extraction Kit (Omega Bio-tek, Cat. No. D2500-02), we obtained libraries with insert sizes of about 300 bp. Finally, PE150 sequencing was performed using the Illumina NovaSeq6000 system at Berry Genomics, Beijing.

### BPS and indel mutation analysis

For the Illumina sequencing data, the 2 × 150 bp paired-end reads were first trimmed by Fastp (v-1.0) (76) to remove adapters and low-quality reads. Then the clean reads were mapped to the reference genome (NZ_CP066804.1 for chromosome and NZ_CP066805.1 for chromid in *P*. sp. LC0214; NZ_CP011039.1 for chromosome and NZ_CP011040.1 for chromid in *P*. sp. JCM12884^T^), using the “mem” function in Burrows–Wheeler Aligner (v-0.7.17) (77). The mapped reads were in SAM format and transformed into BAM format by SAMtools (v-1.9) (78). We used the HaplotypeCaller of Genome Analysis Toolkit (GATK, v-4.1.2.0) (80–82) with standard hard filters to identify the BPSs and indels. MA lines with low coverage (less than 20×) and cross-contamination of sequenced lines were removed. The mutation rates (μ) of BPSs and indels were calculated with the formula as follows:


$$\mu =\frac{m}{\sum_{1}^{n}N\times T}$$

Here, n is the number of MA lines. The number of mutations for all MA lines, the analyzed sites for each MA line, and the total cell divisions during the transfers are denoted by m, N, and T. The context-dependent mutation rates were analyzed using the method proposed by Long et al. (83).

A/T mutation bias was calculated by $\frac{\;\mu_{G:C\to A:T}+\;\mu_{G:C\to T:A}}{\mu_{A:T\to G:C}+\;\mu_{A:T\to C:G}}$, and the transition to transversion ratios (ts/tv; n is the total number of MA lines) with the following formula:


$$\frac{\sum_{1}^{n}\text{transitions}}{\sum_{1}^{n}\text{transversions}}$$

## Data Availability

All FASTQ sequences are available at NCBI BioProject PRJNA1128854. The NCBI SRA accession numbers of *P*. sp. LC0214 MA lines are SAMN42109187 to SAMN42109308. The NCBI SRA accession numbers of *P*. sp. JCM12884^T^ MA lines are SAMN42121910 to SAMN42122051.

## SUPPLEMENTAL MATERIAL

Supplemental figures spectrum.02127-24-s0001.pdf Fig. S1 to S6.

Supplemental material spectrum.02127-24-s0002.pdf Supplemental Materials and Methods.

Supplemental tables spectrum.02127-24-s0003.xlsx Tables S1 to S8.

ASM does not own the copyrights to Supplemental Material that may be linked to, or accessed through, an article. The authors have granted ASM a non-exclusive, world-wide license to publish the Supplemental Material files. Please contact the corresponding author directly for reuse.
