# Supplementary material for: Mutational features of chromids and chromosomes in Pseudoalteromonas provide new insights into the evolution of secondary replicons
Source: Microbiol Spectr. 2025 Mar 25;13(5):e02127-24. doi: 10.1128/spectrum.02127-24 (PMC12053903; doi:10.1128/spectrum.02127-24)
Supplement: Supplemental figures — Fig. S1 to S6. [file spectrum.02127-24-s0001.pdf]

Supplementary Figures

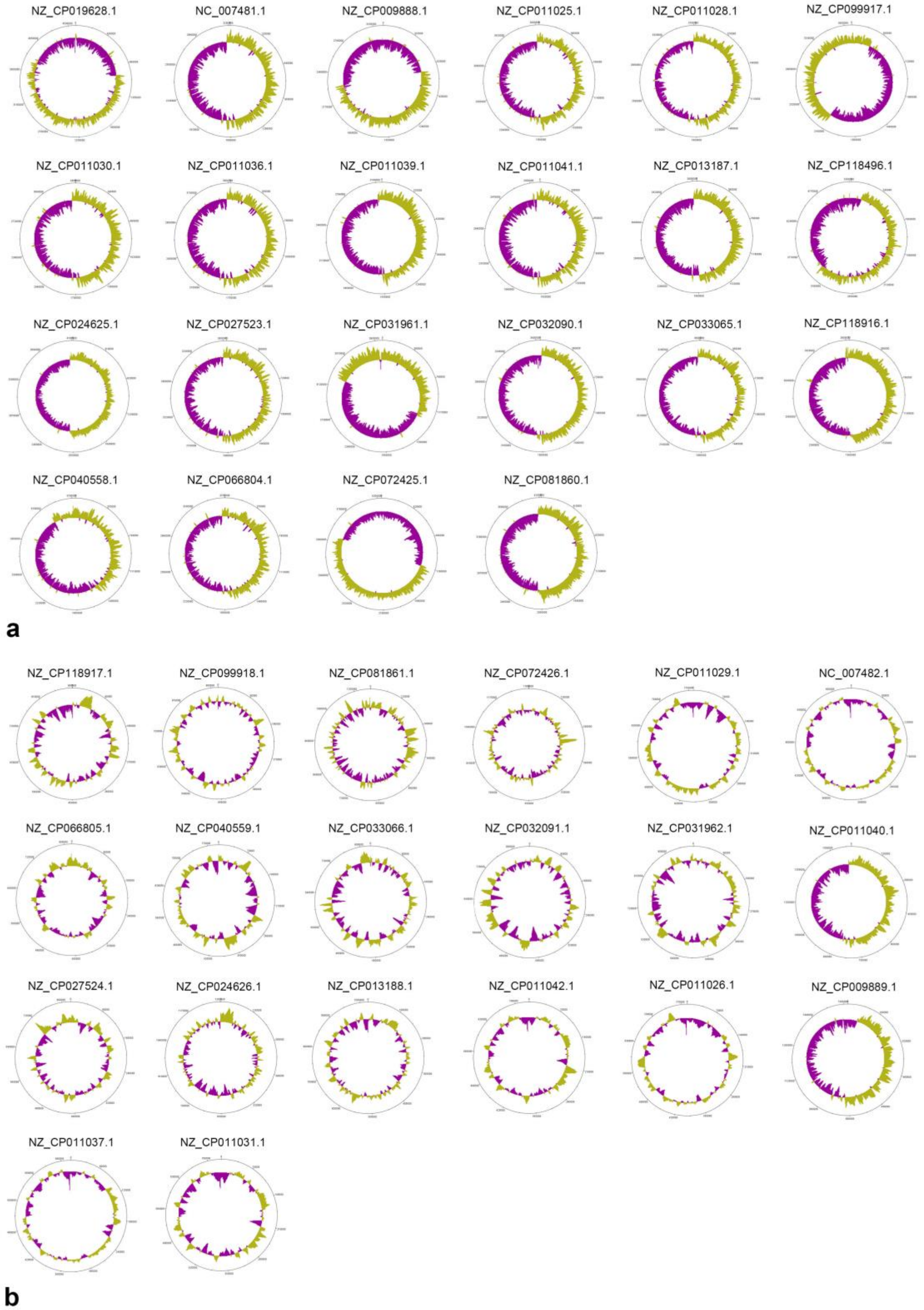

FIG S1 GC skew analysis of chromosomes and chromids of *Pseudoalteromonas* species. a GC skew of chromosomes. **b** GC skew of chromids.

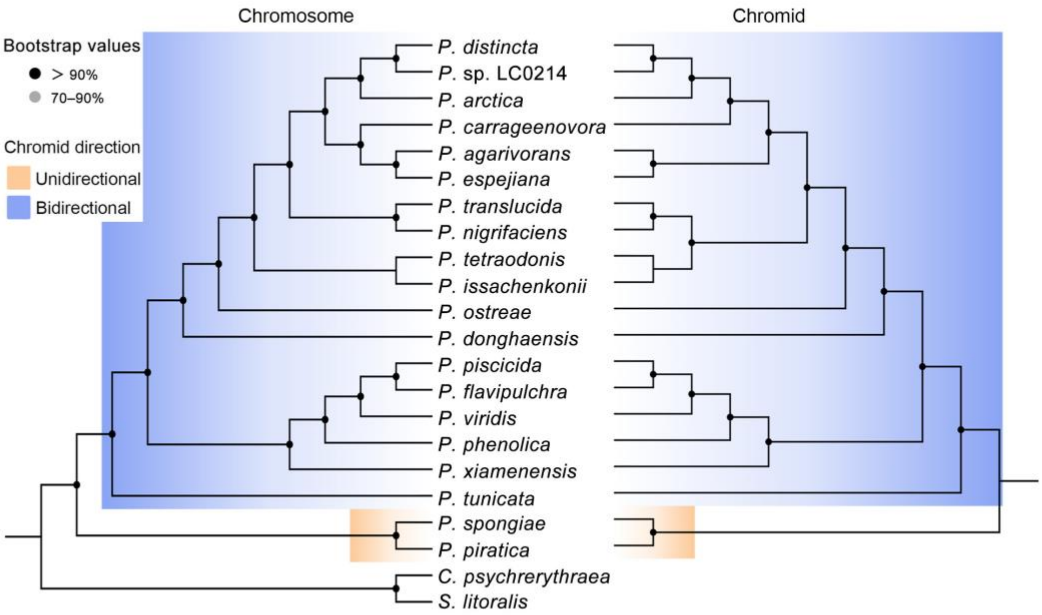

FIG S2 Phylogenetic tree of the chromosome and chromid in *Pseudoalteromonas* genus constructed using the maximum likelihood method. Phylogenetic trees were constructed with the amino acid sequences of single-copy orthologous genes of the chromosome and chromid, respectively. The best model is LG+F+R6 as determined by AIC and BIC tests in chromosome phylogenetic tree, and the best model is LG+F+R5 as determined by AIC and BIC tests in chromid phylogenetic tree. Bootstrap values are greater than 90%.

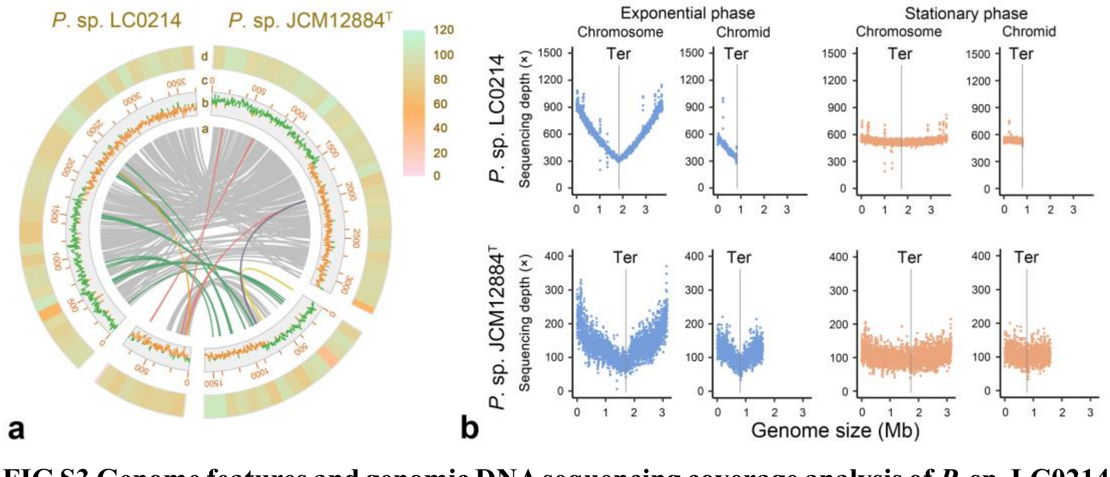

FIG S3 Genome features and genomic DNA sequencing coverage analysis of *P.* sp. LC0214 and *P.* sp. JCM12884^{T}. a Genome features of *P*. sp. LC0214 and *P.* sp. JCM12884^{T}. a-d circles represent gene collinearity, GC skew, replicon scale, and gene density. **b** Genomic DNA sequencing depth analysis of *P*. sp. LC0214 and *P.* sp. JCM12884^{T} at exponential and stationary growth phases, respectively.

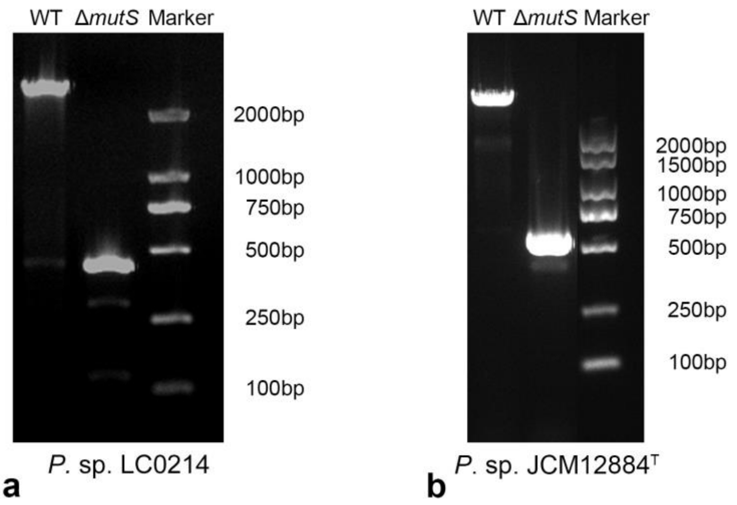

FIG S4 Colony PCR and gel electrophoresis. a *mutS* PCR products of the wild-type and Δ*mutS* in *P*. sp. LC0214. **b** *mutS* PCR products of the wild-type and Δ*mutS* in *P.* sp. JCM12884^{T}.

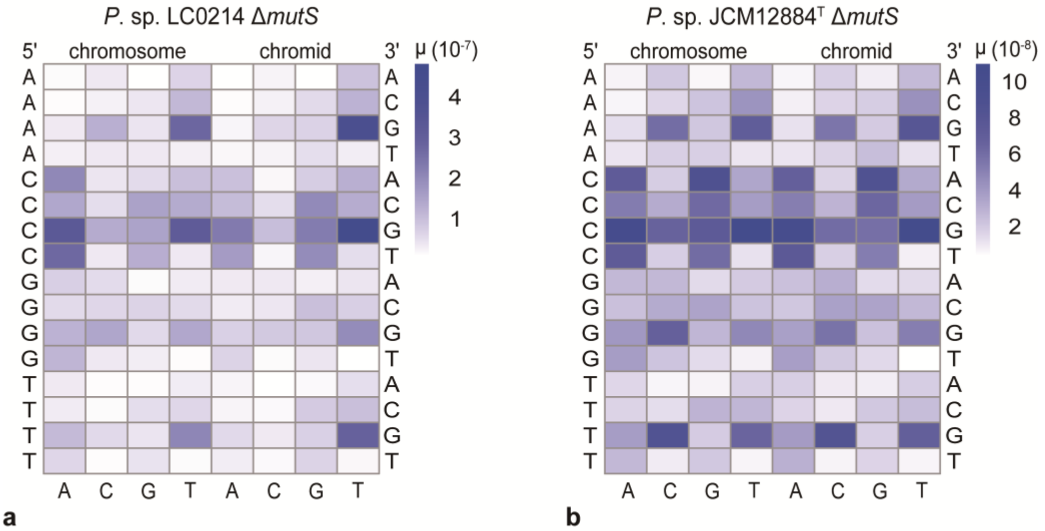

FIG S5 BPS Context-dependent mutation rates of *P.* sp. LC0214 and *P.* sp. JCM12884^{T} Δ*mutS* MA lines. a Chromosome and chromid of *P*. sp. LC0214. **b** Chromosome and chromid of *P.* sp. JCM12884^{T}. The heatmaps show the mutation rate of each triplet (the bases on the bottom are focal bases, the 5' flanking nucleotides and the 3' flanking nucleotides are on the left and right respectively). μ on the scale represents the context-dependent mutation rate (10^{-7} per nucleotide site per cell division for *P*. sp. LC0214 and 10^{-8} per nucleotide site per cell division for *P.* sp. JCM12884^{T}).

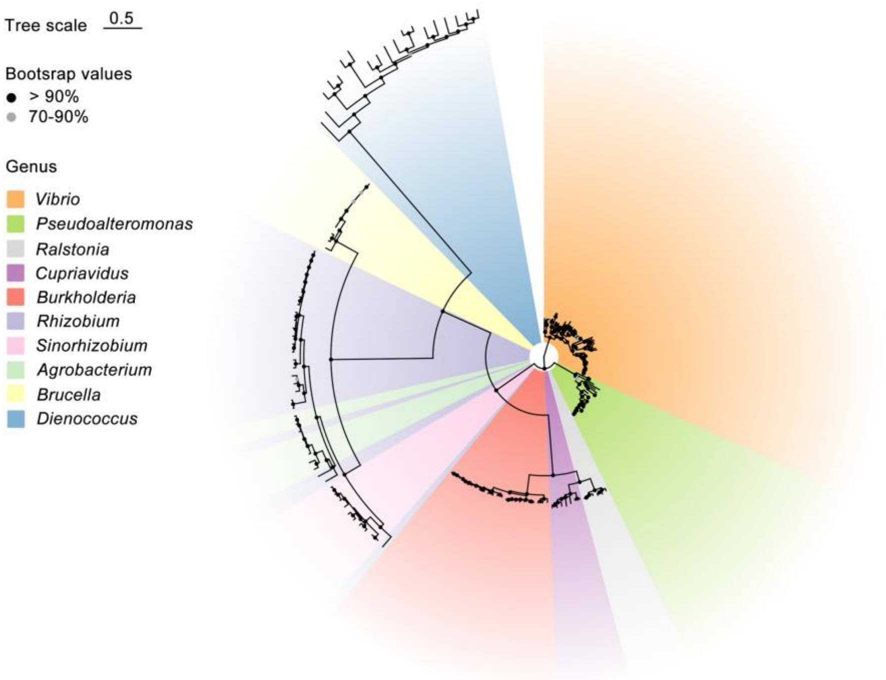

FIG S6 Species phylogenetic tree of 193 multi-replicon species constructed using the maximum likelihood method. Species phylogenetic tree was constructed with the amino acid sequences of single-copy orthologous genes. The best model is **LG+F+R10** as determined by AIC and BIC tests.
