# Supplementary material for: Mutational features of chromids and chromosomes in Pseudoalteromonas provide new insights into the evolution of secondary replicons
Source: Microbiol Spectr. 2025 Mar 25;13(5):e02127-24. doi: 10.1128/spectrum.02127-24 (PMC12053903; doi:10.1128/spectrum.02127-24)
Supplement: Supplemental material — s and Methods. [file spectrum.02127-24-s0002.pdf]

Materials and methods

1. Analyses of the genomic sequence characteristics of *Pseudoalteromonas* replicons

With the continuous enrichment of whole-genome sequencing data, the availability of many complete bacterial genome sequences makes it feasible that we can now examine on a broad scale the characteristics of the different types of replicons in a genome. To analyze replicon sequence characteristics of *Pseudoalteromonas* species, we downloaded the whole-genome sequences of the 22 fully assembled and annotated *Pseudoalteromonas* species from the NCBI database (https://www.ncbi.nlm.nih.gov/datasets/taxonomy/53246/) (NCBI Genome data update date October, 2023). Among the 22 species, 20 are multi-replicon and two are single-replicon species, respectively (Table S1).

We firstly counted the G/C base number of each chromosome and chromid (20 species contain chromids) within the 22 species and calculated the GC content by dividing the G/C base number by the total base number of the replicon. As the trinucleotide composition frequency of genomic sequences is an important feature of genomes for genome evolution, we calculated the trinucleotide composition frequency of the chromosomes and chromids of the 22 species. We calculated the frequency of 64 trinucleotides from the first base of the replicon sequence with self-written script, with a window size of 3 bp and a step size of 1 bp.

We counted the number of CDSs in each chromosome and chromid and calculated the CDS density by dividing the number of CDSs by the total base number of the replicon. To compare the selection intensity of genes on chromosomes and chromids, ParaAT.pl and Paml software were used to calculate the Ka/Ks ratio of 1428 single-copy homologous genes on chromosomes (22 species) and 98 single-copy homologous genes on chromids (20 species). The single-copy homologous genes were obtained using Orthofinder software (v-2.5.4) (1).

2. Phylogenetic tree construction and synteny analyses of the genus *Pseudoalteromonas*

To uncover the phylogenetic relationships within different species of the *Pseudoalteromonas* genus, we downloaded the whole-genome coding region amino acid sequences of the above 22 fully assembled and annotated *Pseudoalteromonas* species, along with the two single-replicon outgroup species, *Saccharobesus litoralis* and *Colwellia psychrerythraea*. Single-copy genes were identified using OrthoFinder (v-2.5.4) (1), and the amino acid sequences of single-copy orthologous genes were aligned using Mafft (v-7.475) software with default parameters (2). IQ-TREE (v-2.1.4) (3) was used to construct the phylogenetic tree based on the maximum likelihood principle after alignment, and the phylogenetic trees were visualized using the Chiplot online website (4). Additionally, we constructed phylogenetic trees based on the single-copy orthologous gene amino acid sequences of the primary replicon (chromosome) and the

secondary replicon (chromid), respectively.

To reveal the gene collinearity relationship between different replicons, we selected several representative species from different evolutionary branches based on the phylogenetic tree. MCScanX (5) software was used to perform gene collinearity analysis. The selected species were as follows: the outgroup single-replicon species *Saccharobesus litoralis*, and the *Pseudoalteromonas* species *P.* sp. JCM12884^T, *P. tunicata*, *P. viridis*, *P. piscicida*, *P. donghaensis*, *P. rhizosphaerae*, *P. tetraodonis*, *P. aliena*, and *P*. sp. LC0214.

3.Replication direction verification of chromids in *P*. sp. LC0214 and *P.* sp. JCM12884^T

To further investigate the evolution and the mutation characteristics of different replicons within the whole genome in the genus *Pseudoalteromonas*, we selected two species, *P.* sp. JCM12884^T and *P*. sp. LC0214 (*P*. sp. LC0214 was also called *P*. sp. LC2018020214 (6)), for further study of their genome mutation features. TBtools software (7) was used first to perform gene collinearity analysis on different replicons of these two species.

In order to verify the replication direction of each replicon in the two species, the cells of each species were cultured in marine LB broth at 25°C and harvested at the exponential phase (~ 4h) and the stationary phase (24 to 40 h). Genomic DNA was extracted using the MasterPure^{TM} Complete DNA & RNA Purification Kit according to the product instructions. A 300-bp insert size library was constructed for each sample, and sequenced with an Illumina Novaseq6000 system with PE150 bp reads. After been filtered by Fastp (v-1.0) (8), the clean reads were mapped to replicons using Burrows-Wheeler Aligner (v-0.7.17) (9). The coverage of every base-pair was calculated using the mpileup subprogram in SAMtools (10). The coverage data were further grouped in bins of 1 kbp.

4. Construction of *P*. sp. LC0214 and *P.* sp. JCM12884^T MMR -deficient strains

We constructed MMR-deficient strains (Δ*mutS*) of *P*. sp. LC0214 and *P.* sp. JCM12884^T using markerless deletion of the *mutS* gene with the principle of homologous recombination (11). The method introduced a fragment of upstream and downstream homology arms of *mutS* gene into the strains, replacing the wild-type *mutS* gene sequence via homologous recombination, thereby generating MMR-deficient knockout strains.

5. Mutation accumulation (MA) procedures

To explore the whole-genome mutational features of different types of replicons in *P*. sp. LC0214 and *P.* sp. JCM12884^T, we performed MA experiments on the four ancestral strains (*P.* sp. LC0214 WT/Δ*mutS*, *P.* sp. JCM12884^T WT/Δ*mutS*). For each strain, to initiate the MA lines,

cells from an ancestral colony were plated onto marine LB agar plates. Sixty MA lines were initiated of *P*. sp. LC0214 WT and Δ*mutS* strains; and seventy MA lines were initiated of *P.* sp. JCM12884^T WT and Δ*mutS* strains. All 260 MA lines were cultured on marine LB agar (Solarbio, Cat. No.: L8290) at 25 °C, with each line being single-colony transferred daily.

The MA experiments lasted 80 to 100 days, during which each WT MA line was transferred for 100 times and each Δ*mutS* MA line was transferred for 80 times on average. In order to estimate the cell divisions (t) between transfers (Num) by the colony-forming-units, we performed serial dilution every ~30 days, by randomly choosing and razor-cutting a single colony from each of the 5 lines for the WT and the Δ*mutS* MA lines, based on the formula:

$$t = log_2(Num)$$

6. DNA Extraction, Library Construction, and Genome Sequencing

After the last transfer, we picked a single colony for each final MA line as well as the ancestral line for each strain and cultured them in marine LB broth (Solarbio, Cat. No.: L8291) overnight at 25 °C. We then extracted genomic DNA using the MasterPure™ Complete DNA and RNA Purification Kit (Lucigen, Cat No.: MC85200). Short-read libraries of DNA that met the concentration and quality requirements were constructed using an optimized protocol for the TruePrep® DNA Library Prep Kit V2 for Illumina (Vazyme, Cat. No.: TD501-01) and the TruePrep® Index Kit V3 for Illumina (Vazyme, Cat. No.: TD203). After performing agarose gel electrophoresis and cutting the target bands for recycling with the E.Z.N.A.® Gel Extraction Kit (Omega Bio-tek, Cat. No.: D2500-02), we obtained libraries with insert sizes of about 300 bp. Finally, PE150 sequencing was performed using Illumina NovaSeq6000 system at Berry Genomics, Beijing.

7. BPS and Indel Mutation Analysis

For the Illumina sequencing data, the 2 × 150 bp paired-end reads were first trimmed by Fastp (v-1.0) (8) to remove adapters and low-quality reads. Then the clean reads were mapped to the reference genome (NZ_CP066804.1 for chromosome and NZ_CP066805.1 for chromid in *P*. sp. LC0214; NZ_CP011039.1 for chromosome and NZ_CP011040.1 for chromid in *P.* sp. JCM12884^T), using the "mem" function in Burrows–Wheeler Aligner (v-0.7.17) (9). The mapped reads were in SAM format and transformed into BAM format by SAMtools (v-1.9) (10). We used the HaplotypeCaller of Genome Analysis Toolkit (GATK, v-4.1.2.0) (12-14) with standard hard filters to identify the BPSs and indels. MA lines with low coverage (less than 20×) and cross-contamination of sequenced lines were removed. The mutation rates (μ) of BPSs and indels were calculated with the formula as follow:

$$\mu = \frac{m}{\sum_1^n \mathrm{N} \times \mathrm{T}}$$

Here, n is the number of MA lines. The number of mutations for all MA lines, the analyzed sites for each MA line, and the total cell divisions during the transfers are denoted by m, N, and T. The context-dependent mutation rates were analyzed using the method proposed by Long et al.(15).

The mutation bias m was calculated by $m = \mu_{G:C\rightarrow A:T+G:C\rightarrow T:A}/\mu_{A:T\rightarrow G:C+A:T\rightarrow C:G}$, and the transition to transversion ratios (ts/tv; n is the total number of MA lines) with the following formula:

$$\frac{\sum_1^n \text{transitions}}{\sum_1^n \text{transversions}}$$

Reference

1. Emms DM, Kelly S. 2019. OrthoFinder: phylogenetic orthology inference for comparative genomics. Genome Biology 20:1–14.
2. Katoh K, Standley DM. 2013. MAFFT multiple sequence alignment software version 7: improvements in performance and usability. Molecular Biology and Evolution 30:772–780.
3. Minh BQ, Schmidt HA, Chernomor O, Schrempf D, Woodhams MD, Von Haeseler A, Lanfear R. 2020. IQ-TREE 2: new models and efficient methods for phylogenetic inference in the genomic era. Molecular Biology and Evolution 37:1530–1534.
4. Xie J, Chen Y, Cai G, Cai R, Hu Z, Wang H. 2023. Tree Visualization By One Table (tvBOT): a web application for visualizing, modifying and annotating phylogenetic trees. Nucleic Acids Research:gkad359.
5. Wang Y, Tang H, DeBarry JD, Tan X, Li J, Wang X, Lee T-h, Jin H, Marler B, Guo H. 2012. MCScanX: a toolkit for detection and evolutionary analysis of gene synteny and collinearity. Nucleic Acids Research 40:e49.
6. Wattanadilokchatkun P, Kayansamruaj P, Pan J. 2021. Complete Genome Sequence of *Pseudoalteromonas* sp. Strain LC2018020214, a Bacterium Isolated from Natural Seawater. Microbiology Resource Announcements 10:10.1128/mra. 00075–21.
7. Chen C, Chen H, Zhang Y, Thomas HR, Frank MH, He Y, Xia R. 2020. TBtools: an integrative toolkit developed for interactive analyses of big biological data. Molecular Plant 13:1194–1202.
8. Chen S, Zhou Y, Chen Y, Gu J. 2018. fastp: an ultra-fast all-in-one FASTQ preprocessor. Bioinformatics 34:i884–i890.
9. Li H. 2013. Aligning sequence reads, clone sequences and assembly contigs with BWA-MEM. arXiv preprint arXiv:13033997.
10. Li H, Handsaker B, Wysoker A, Fennell T, Ruan J, Homer N, Marth G, Abecasis G, Durbin R, Subgroup GPDP. 2009. The sequence alignment/map format and SAMtools. Bioinformatics 25:2078–2079.
11. Wang P, Yu Z, Li B, Cai X, Zeng Z, Chen X, Wang X. 2015. Development of an efficient conjugation-based genetic manipulation system for *Pseudoalteromonas*. Microbial Cell Factories 14:1–11.
12. McKenna A, Hanna M, Banks E, Sivachenko A, Cibulskis K, Kernytsky A, Garimella K,

Altshuler D, Gabriel S, Daly M. 2010. The Genome Analysis Toolkit: a MapReduce framework for analyzing next-generation DNA sequencing data. Genome Research 20:1297–1303.

13. DePristo MA, Banks E, Poplin R, Garimella KV, Maguire JR, Hartl C, Philippakis AA, Del Angel G, Rivas MA, Hanna M. 2011. A framework for variation discovery and genotyping using next-generation DNA sequencing data. Nature Genetics 43:491–498.

14. Van der Auwera GA, Carneiro MO, Hartl C, Poplin R, Del Angel G, Levy-Moonshine A, Jordan T, Shakir K, Roazen D, Thibault J. 2013. From FastQ data to high-confidence variant calls: the genome analysis toolkit best practices pipeline. Current Protocols in Bioinformatics 43:11.10. 1–11.10. 33.

15. Long H, Sung W, Miller SF, Ackerman MS, Doak TG, Lynch M. 2015. Mutation rate, spectrum, topology, and context-dependency in the DNA mismatch repair-deficient *Pseudomonas fluorescens* ATCC948. Genome Biology and Evolution 7:262–271.
